# Supplementary material for: Cross-population coupling of neural activity based on Gaussian process current source densities
Source: PLoS Comput Biol. 2021 Nov 17;17(11):e1009601. doi: 10.1371/journal.pcbi.1009601 (PMC8635346; doi:10.1371/journal.pcbi.1009601)
Supplement: S1 Text — More details on a priori physical models and Gaussian process computational details; additional simulation results to address model mismatch situations. (PDF) [file pcbi.1009601.s001.pdf]

Supplement: Cross-population coupling of neural activity based on Gaussian process current source densities

Natalie Klein[1, 2¤], Joshua H. Siegle[3], Tobias Teichert[4], Robert E. Kass[1, 2, 5*]

1 Department of Statistics and Data Science, Carnegie Mellon University, Pittsburgh, Pennsylvania, USA

2 Machine Learning Department, Carnegie Mellon University, Pittsburgh, Pennsylvania, USA

3 MindScope Program, Allen Institute, Seattle, Washington, USA

4 Departments of Psychiatry and Bioengineering, University of Pittsburgh, Pittsburgh, Pennsylvania, USA

5 Neuroscience Institute, Carnegie Mellon University, Pittsburgh, Pennsylvania, USA

* kass@stat.cmu.edu

¤Current address: Statistical Sciences Group, Los Alamos National Laboratory, Los Alamos, New Mexico, USA

Supporting information (S1 Text)

One-dimensional *a priori* physical model details

For completeness, we give the derivation of the forward model, which was originally proposed in [1]. Substituting the *a priori* cylinder model into the three-dimensional forward model yields

$$\phi(x,y,z) = -\frac{1}{4\pi\varsigma}\int_a^b \int\int_{x^2+y^2\leq R} \frac{g(z')}{\sqrt{(x-x')^2+(y-y')^2+(z-z')^2}}\,dx'\,dy'\,dz'. \tag{S.1}$$

We assume x and y are inside the cylinder (as typically, we assume we observe ϕ at the center of the cylinder). Changing to polar coordinates, we define $r^2 = (x-x')^2 + (y-y')^2$ as the variable radius inside the cylinder and use the

substitution $dx'\,dy'\,dz' = r\,d\theta\,dr\,dz'$ to obtain

$$\phi(x,y,z) = -\frac{1}{4\pi\varsigma}\int_a^b\int_0^R\int_0^{2\pi}\frac{rg(z')}{\sqrt{(z-z')^2+r^2}}\,d\theta\,dr\,dz' \tag{S.2}$$

$$= -\frac{1}{2\varsigma}\int_a^b g(z')\int_0^R\frac{r}{\sqrt{(z-z')^2+r^2}}\,dr\,dz' \tag{S.3}$$

$$= -\frac{1}{2\varsigma}\int_a^b g(z')\left[\sqrt{(z-z')^2+R^2}-\sqrt{(z-z')^2}\right]\,dz'. \tag{S.4}$$

Notice that after integration, this is no longer a function of x or y, so we can simply write

$$\phi(z) = -\frac{1}{2\varsigma}\int_a^b g(z')\left[\sqrt{(z-z')^2+R^2}-\sqrt{(z-z')^2}\right]\,dz'. \tag{S.5}$$

To better understand how R affects the ϕ, we can factor out R:

$$\phi(z) = -\frac{R}{2\varsigma}\int_a^b g(z')\underbrace{\left[\sqrt{\left(\frac{r}{R}\right)^2+1}-\sqrt{\left(\frac{r}{R}\right)^2}\right]}_{a(z,z';R)}\,dz' \tag{S.6}$$

where $r = z - z'$ and $a(z, z'; R)$ is a weight function with a maximum value of 1 when $r = 0$. Typically, we are interested only in the relative magnitude of the CSD in space and time so the scalar $\frac{R}{2\varsigma}$ can be ignored.

Two-dimensional *a priori* physical model details

As mentioned in the text, certain choices of two-dimensional *a priori* physical models may lead to singularities in the forward model. To avoid the singularity, we assume that there is some region of zero CSD surrounding the probe, which is parameterized by τ. As shown by [2], substituting the *a priori* model into the three-dimensional forward model yields

$$\begin{aligned}\phi(x,y,z) &= -\frac{1}{4\pi\varsigma}\int_{a_z}^{b_z}\int_{a_y}^{b_y}\int_{\tau\le x\le R+\tau}\frac{g(y',z')}{\sqrt{(x-x')^2+(y-y')^2+(z-z')^2}}\,dx'\,dy'\,dz' \\ &= -\frac{1}{4\pi\varsigma}\int_{a_z}^{b_z}\int_{a_y}^{b_y}\int_{\tau}^{R+\tau}\frac{g(y',z')}{\sqrt{(x-x')^2+m^2}}\,dx'\,dy'\,dz' \\ &= -\frac{1}{4\pi\varsigma}\int_{a_z}^{b_z}\int_{a_y}^{b_y} g(y',z')\int_{\tau}^{R+\tau}\frac{1}{\sqrt{(x-x')^2+m^2}}\,dx'\,dy'\,dz'\end{aligned}$$

where $m = \sqrt{(y-y')^2 + (z-z')^2}$. Since we are interested in modeling the LFP at the face of the probe ($x = 0$), let $x = 0$ and integrate over x':

$$\begin{aligned}\phi(0, y, z) &= -\frac{1}{4\pi\varsigma}\int_{a_z}^{b_z}\int_{a_y}^{b_y} g(y', z')\int_{\tau}^{R+\tau}\frac{1}{\sqrt{(x')^2 + m^2}}\,dw\,dy'\,dz' \\ &= -\frac{1}{4\pi\varsigma}\int_{a_z}^{b_z}\int_{a_y}^{b_y} g(y', z')\Big[\log(R+\tau+\sqrt{(R+\tau)^2 + (y-y')^2 + (z-z')^2} \\ &\qquad - \log(\tau + \sqrt{\tau^2 + (y-y')^2 + (z-z')^2}\Big]\,dy'\,dz'.\end{aligned}$$

We will write the LFP as $\phi(y, z)$ where implicitly $x = 0$ when using the forward model. As in the one-dimensional case, $\frac{1}{4\pi\varsigma}$ may be dropped if one is only interested in the relative variation of the CSD across space and time (and in many cases, ς may not be known).

Computational details for Gaussian process

Let $\mathbf{K}^s \in \mathbb{R}^{M\times M}$ be the LFP spatial covariance evaluated at the locations of the observed LFPs and $\mathbf{K}^t \in \mathbb{R}^{T\times T}$ be the temporal covariance evaluated at the observed time points; they are functions of $\boldsymbol{\theta}$ (including the forward model parameter R which is part of the LFP spatial covariance function through the forward operator; the computation of $\mathbf{K}^s$ is discussed in the next section). Let $\tilde{\boldsymbol{\phi}}^{(r)} \in \mathbb{R}^{M\times T}$ represent the matrix of observed LFPs on trial n (with N total trials). The traditional expression for the log marginal likelihood (excluding terms that don't depend on $\boldsymbol{\theta}$) takes the form

$$\log\mathcal{L}(\boldsymbol{\theta}) = -\frac{1}{2}\sum_{n=1}^{N}\log\left(\left|\mathbf{K}^s\otimes\mathbf{K}^t + \sigma^2\mathbf{I}\right|\right) + \mathrm{vec}\left(\tilde{\boldsymbol{\phi}}^{(r)}\right)^T\left[\mathbf{K}^s\otimes\mathbf{K}^t + \sigma^2\mathbf{I}\right]^{-1}\mathrm{vec}\left(\tilde{\boldsymbol{\phi}}^{(r)}\right).$$

However, this form relies on inversion of an $MT \times MT$ matrix which is clearly problematic for typical M and T observed in real data, so we instead leverage the special structure present in the matrix. In particular, we use the eigendecomposition of the covariance matrices, $\mathbf{K}^s = \mathbf{Q}_s\boldsymbol{\Lambda}_s\mathbf{Q}_s^T$ and $\mathbf{K}^t = \mathbf{Q}_t\boldsymbol{\Lambda}_t\mathbf{Q}_t^T$, where $\boldsymbol{\Lambda}_s$ and $\boldsymbol{\Lambda}_t$ are

diagonal matrices. This is useful because of the following identity:

$$\begin{aligned}\left[\mathbf{K}^s \otimes \mathbf{K}^t + \sigma^2 \mathbf{I}\right]^{-1} &= \left[\mathbf{Q}_s \mathbf{\Lambda}_s \mathbf{Q}_s^T \otimes \mathbf{Q}_t \mathbf{\Lambda}_t \mathbf{Q}_t^T + \sigma^2 \mathbf{I}\right]^{-1} \\ &= \left[(\mathbf{Q}_s \otimes \mathbf{Q}_t)(\mathbf{\Lambda}_s \otimes \mathbf{\Lambda}_t)(\mathbf{Q}_s^T \otimes \mathbf{Q}_t^T) + \sigma^2 \mathbf{I}\right]^{-1} \\ &= \left[(\mathbf{Q}_s \otimes \mathbf{Q}_t)(\mathbf{\Lambda}_s \otimes \mathbf{\Lambda}_t)(\mathbf{Q}_s^T \otimes \mathbf{Q}_t^T) + \sigma^2 (\mathbf{Q}_s \otimes \mathbf{Q}_t)(\mathbf{Q}_s \otimes \mathbf{Q}_t)^{-1}\right]^{-1} \\ &= \left[(\mathbf{Q}_s \otimes \mathbf{Q}_t)(\mathbf{\Lambda}_s \otimes \mathbf{\Lambda}_t)(\mathbf{Q}_s^T \otimes \mathbf{Q}_t^T) + (\mathbf{Q}_s \otimes \mathbf{Q}_t)(\sigma^2 \mathbf{I})(\mathbf{Q}_s^T \otimes \mathbf{Q}_t^T)\right]^{-1} \\ &= \left[(\mathbf{Q}_s \otimes \mathbf{Q}_t)(\mathbf{\Lambda}_s \otimes \mathbf{\Lambda}_t + \sigma^2 \mathbf{I})(\mathbf{Q}_s^T \otimes \mathbf{Q}_t^T)\right]^{-1} \\ &= (\mathbf{Q}_s \otimes \mathbf{Q}_t)\left[\mathbf{\Lambda}_s \otimes \mathbf{\Lambda}_t + \sigma^2 \mathbf{I}\right]^{-1}(\mathbf{Q}_s^T \otimes \mathbf{Q}_t^T)\end{aligned}$$

which relies on properties of the Kronecker product and the orthonormality of $(\mathbf{Q}_s \otimes \mathbf{Q}_t)$. This implies that after eigendecomposition, the inversion of the matrix reduces to inversion of a diagonal matrix.

Let $\mathbf{D} = \mathbf{\Lambda}_s \otimes \mathbf{\Lambda}_t + \sigma^2 \mathbf{I}$ be a diagonal matrix, and let $\mathbf{q}$ be an MT-vector with elements $1/D_{ii}$. Note that a low-rank Gaussian process could be implemented by using truncated eigendecompositions [4]. Using the eigendecomposition and properties of Kronecker products (as shown in more detail in [3]), the log marginal likelihood may be rewritten:

$$\log \mathcal{L}(\boldsymbol{\theta}) = -\frac{N}{2}\sum_{i=1}^{MT}\log(D_{ii}) - \frac{1}{2}\sum_{n=1}^{N}\sum_{i=1}^{MT}\left[\text{vec}\left(\mathbf{Q}_s^T \tilde{\boldsymbol{\phi}}^{(r)} \mathbf{Q}_t\right) \circ \text{vec}\left(\mathbf{Q}_s^T \tilde{\boldsymbol{\phi}}^{(r)} \mathbf{Q}_t\right) \circ \mathbf{q}\right]_i$$

where the Hadamard product $\circ$ indicates elementwise multiplication and $\mathbf{Q}_t$, $\mathbf{Q}_s$, $\mathbf{D}$, and $\mathbf{q}$ depend on $\boldsymbol{\theta}$. This form is faster and more stable to compute as it avoids direct inversion of an $MT \times MT$ matrix which has computational complexity $O(M^3T^3)$. Because eigendecomposition is of complexity $O(M^3)$ and $O(T^3)$ for the spatial and temporal covariance matrices, respectively, the computational complexity of one likelihood function evaluation is instead $O(M^3 + T^3 + MT)$ for both eigendecompositions and the inversion of a diagonal matrix of size $MT \times MT$.

Given fixed $\boldsymbol{\theta}$, the log marginal likelihood may also be optimized over mean function parameters $\boldsymbol{\gamma}$; here we show the likelihood assuming a shared mean function across trials, though per-trial mean parameters could also be used (and if trials were assumed independent, this would result in a separate log marginal likelihood for each trial). Let $\boldsymbol{\mu} \in \mathbb{R}^{M \times T}$ be the mean function evaluated at the observed LFP spatial and temporal

points (where this function depends on $\boldsymbol{\gamma}$). We first calculate the inverse covariance matrix as

$$\boldsymbol{\Sigma}^{-1} = (\mathbf{Q}_s \otimes \mathbf{Q}_t)\,\mathrm{diag}(\mathbf{q})\,(\mathbf{Q}_s \otimes \mathbf{Q}_t)^T$$

and calculate the mean of the LFPs across trials as $\bar{\mathbf{y}} = \frac{1}{N_r}\sum_{r=1}^{N_r} \tilde{\boldsymbol{\phi}}^{(r)}$. Then we use the following (rescaled) log marginal likelihood:

$$\log \mathcal{L}(\boldsymbol{\gamma}) = \mathrm{vec}(\boldsymbol{\mu})^T \boldsymbol{\Sigma}^{-1}\mathrm{vec}(\bar{\mathbf{y}}) - \frac{1}{2}\mathrm{vec}(\boldsymbol{\mu})^T \boldsymbol{\Sigma}^{-1}\mathrm{vec}(\boldsymbol{\mu}).$$

Numerical integration in computing covariance matrices

To compute the spatial LFP covariance matrix, the forward model must be applied to the CSD covariance function and evaluated at the observed LFP spatial locations. We will assume that the integral is approximated using a standard numerical integration scheme of the form

$$\int_a^b f(u)\,du \approx \sum_i w_i f(u_i)$$

where w_i are weights that may depend on a quadrature scheme or the distance between the u_i points. In the case of a covariance function for the LFP, we apply the forward model integral equation to both inputs of the covariance function, so that the function evaluated at a single pair of inputs (x, x') takes the form

$$\int_a^b \int_a^b b(x-u)b(x'-v)k(u,v)\,du\,dv \approx \sum_i \sum_j w_i^u w_j^v b(x-u_i)b(x'-v_j)k(u_i,v_j) \quad \text{(S.7)}$$

where k is the CSD covariance function and b are the forward model weights. Assume we want to evaluate the LFP covariance matrix at all pairs of locations from two vectors $\mathbf{x} = [x_1, ..., x_m]$ and $\mathbf{x}' = [x_1, ..., x_n]$, and assume we have vectors $\mathbf{u} = [u_1, ..., u_c]$ and

$\mathbf{v} = [v_1, ..., v_d]$ spanning the ranges of the integrals. Define the following matrices:

$$\mathbf{A} = \begin{bmatrix} w_1^u b(x_1 - u_1) & \cdots & w_c^u b(x_1 - u_c) \\ w_1^u b(x_2 - u_1) & \cdots & w_c^u b(x_2 - u_c) \\ \vdots & \vdots & \vdots \\ w_1^u b(x_m - u_1) & \cdots & w_c^u b(x_m - u_c) \end{bmatrix} \in \mathbb{R}^{m \times c},$$

$$\mathbf{B} = \begin{bmatrix} w_1^v b(x_1' - v_1) & \cdots & w_1^v b(x_n' - v_1) \\ w_2^v b(x_1' - v_2) & \cdots & w_2^v b(x_n' - v_2) \\ \vdots & \vdots & \vdots \\ w_d^v b(x_1' - v_d) & \cdots & w_d^v b(x_n' - v_d) \end{bmatrix} \in \mathbb{R}^{d \times n},$$

$$\mathbf{K} = \begin{bmatrix} k(u_1, v_1) & \cdots & k(u_1, v_d) \\ k(u_2, v_1) & \cdots & k(u_2, v_d) \\ \vdots & \vdots & \vdots \\ k(u_c, v_1) & \cdots & k(u_c, v_d) \end{bmatrix} \in \mathbb{R}^{c \times d}.$$

Then the LFP spatial covariance may be computed as $\mathbf{AKB}$. Similarly, the spatial cross-covariance between the LFP and the CSD may be computed as $\mathbf{AK}$. We found that using simple integration rules (midpoint or trapezoid rule) worked well given large enough c and d. Notice that the multiplication $\mathbf{AKB}$ must be done prior to the eigendecomposition, or else the orthonormality need to establish the key identity for computing the matrix inverse is not preserved.

The scheme described above also applies directly to the two-dimensional case. To evaluate a single element of the covariance matrix at spatial locations (x_1, x_2) and (x_1', x_2'), where single subscripts now represent dimension indexing, we evaluate the integral

$$\int_{a_2}^{b_2} \int_{a_2}^{b_2} \int_{a_1}^{b_1} \int_{a_1}^{b_1} b(x_1 - u_1, x_2 - u_2) b(x_1' - v_1, x_2' - v_2) k(u_1, u_2, v_1, v_2) \, du_1 \, dv_1 \, du_2 \, dv_2.$$

Now assuming $\mathbf{u}$ and $\mathbf{v}$ are two-dimensional grids indexed as $u_{k,i}$ and $v_{k,j}$ where k is the dimension index in $\{1, 2\}$ and i, j are the element indices, the integral can be

approximated as

$$\sum_i \sum_j w_i^u w_j^v b(x_1 - u_{1,i}, x_2 - u_{2,i}) b(x_1' - v_{1,j}, x_2' - v_{2,j}) k(u_{1,i}, u_{2,i}, v_{1,j}, v_{2,j})$$

which is again a double sum and can be written in the form **AKB**.

In evaluating the likelihood and making predictions with the Gaussian process, the spatial covariance matrix must be calculated using the numerical integration schemes discussed in this section. Given n_s spatial points in the integration grid, computing the spatial covariance matrix requires n_s^2 calculations. While n_s is chosen by the user, a small value leads to inaccurate numerical integration. In addition, while moderate n_s may be reasonable for one-dimensional GPCSD, two-dimensional GPCSD generally requires larger n_s because it is the total number of points in a two-dimensional grid, so is typically roughly quadratic in the number of points required for one-dimensional integration. If the forward model parameter R and the Gaussian process parameters were known, the spatial covariance matrix could be computed once with a cost of $O(n_s^2)$ and the computational complexity of following likelihood evaluations would be $O(M^3 + T^3 + MT)$ where M is the number of observed spatial points and T is the number of observed time points. However, the numerical integration needed to compute the spatial covariance matrix must be repeated each time the spatial covariance or forward model parameters change, leading to $O(n_s^2 + M^3 + T^3 + MT)$ for each likelihood evaluation as those parameters are varied.

Additional simulation results

In addition to simulating from a GPCSD model then fitting a GPCSD model with the same model form, we also investigated simulating from a GPCSD model and fitting a mis-specified GPCSD model. We looked at two cases; for each case, we again generated 50 trials each with 60 time points and 24 spatial locations (similar to the auditory LFP probe).

First, we fit a GPCSD model with only a squared exponential temporal covariance plus LFP white noise to data generated from a squared exponential (lengthscale 20, variance 1.5), plus Matérn (lengthscale 2, variance 0.2), plus LFP white noise. The

average MSE across 50 trials was 0.01, which is orders of magnitude higher than we found using a correctly specified model in the main text. Visually, the estimated CSD captured the overall slow-timescale trend, but failed to capture fast non-white-noise fluctuations. These results suggest that models that are not flexible enough may fail to fit the data well. While lack of fit (in the LFP space) can be used to diagnose this issue, and models with varying numbers of components can be compared by their negative log likelihood values, it may be difficult to pin down the exact number of components that best explains a particular data set. That is, diagnostics can suggest a more flexible model is needed, but in the spirit of model parsimony, decisively selecting a minimal number of components may require a subjective judgement.

Second, we fit a GPCSD model with two components (squared exponential and Matérn) to data generated using three components (fast squared exponential with lengthscale 10 and variance 0.5, slow squared exponential with lengthscale 100 and variance 0.1, and Matérn with lengthscale 2 and variance 0.2). In this case, the average MSE across trials was 7.4×10^{-5}, similar to the error with a correctly specified model in the main text. This case study demonstrates that if the fitted model form is flexible enough, it can achieve a good fit to the data even if the underlying true generating process contained more components (which may be difficult to disentangle based on real data if, for instance, their temporal spectral properties overlap).

References

1. Pettersen KH, Devor A, Ulbert I, Dale AM, Einevoll GT. Current-source density estimation based on inversion of electrostatic forward solution: effects of finite extent of neuronal activity and conductivity discontinuities. Journal of Neuroscience Methods. 2006;154(1-2):116–133.

2. Łeski S, Pettersen KH, Tunstall B, Einevoll GT, Gigg J, Wójcik DK. Inverse current source density method in two dimensions: inferring neural activation from multielectrode recordings. Neuroinformatics. 2011;9(4):401–425.

3. Saatci Y. Scalable inference for structured Gaussian process models; 2012.

4. Solin A, Särkkä S. Hilbert space methods for reduced-rank Gaussian process regression. arXiv preprint arXiv:14015508. 2014;.
