# Supplementary figures and images for: Cross-population coupling of neural activity based on Gaussian process current source densities
Source: PLoS Comput Biol. 2021 Nov 17;17(11):e1009601. doi: 10.1371/journal.pcbi.1009601 (PMC8635346; doi:10.1371/journal.pcbi.1009601)

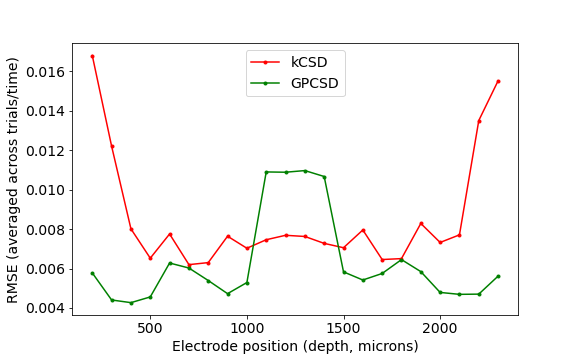

Supplement: S1 Fig — Average MSE (across trials/time) for the simulation study in the main text (with data generated from a GPCSD model). While our results suggested that kCSD and GPCSD overall perform similarly for this data, kCSD appears to have higher error at the edges while GPCSD has higher error in the center of the array. (PNG) [file pcbi.1009601.s002.png]
